# *RSRC1* loss-of-function variants cause mild to moderate autosomal recessive intellectual disability

**DOI:** 10.1093/brain/awaa070

**Published:** 2020-03-30

**Authors:** Marcello Scala, Majid Mojarrad, Saima Riazuddin, Karlla W Brigatti, Zineb Ammous, Julie S Cohen, Heba Hosny, Muhammad A Usmani, Mohsin Shahzad, Sheikh Riazuddin, Valentina Stanley, Atiye Eslahi, Richard E Person, Hasnaa M Elbendary, Anne M Comi, Laura Poskitt, Vincenzo Salpietro, Queen Square Genomics, Jill A Rosenfeld, Katie B Williams, Dana Marafi, Fan Xia, Marta Biderman Waberski, Maha S Zaki, Joseph Gleeson, Erik Puffenberger, Henry Houlden, Reza Maroofian

**Affiliations:** a1 UCL Queen Square Institute of Neurology, University College London, London, UK; a2 Department of Neurosciences, Rehabilitation, Ophthalmology, Genetics, Maternal and Child Health, University of Genoa, Genoa, Italy; a3 Pediatric Neurology and Muscular Diseases Unit, IRCCS Istituto Giannina Gaslini, Genoa, Italy; a4 Department of Medical Genetics, Faculty of Medicine, Mashhad University of Medical Sciences, Mashhad, Iran; a5 Medical Genetics Research Center, Mashhad University of Medical Sciences, Mashhad, Iran; a6 Genetic Center of Khorasan Razavi, Mashhad, Iran; a7 Department of Otorhinolaryngology Head and Neck Surgery, School of Medicine, University of Maryland, Baltimore, MD 21201, USA; a8 Clinic for Special Children, Strasburg, PA, USA; a9 Community Health Clinic, Topeka, IN, USA; a10 Departments of Neurology and Pediatrics, Kennedy Krieger Institute, Johns Hopkins Medical Institutions, Baltimore, MD, USA; a11 National Institute of Neuromotor System, Cairo, Egypt; a12 Center for Genetic Diseases, Shaheed Zulfiqar Ali Bhutto Medical University, Pakistan Institute of Medical Sciences, Islamabad, Pakistan; a13 National Centre of Excellence in Molecular Biology, University of the Punjab, Lahore 53700, Pakistan; a14 Department of Neuroscience, Rady Children’s Institute for Genomic Medicine, Howard Hughes Medical Institute, University of California, San Diego, CA, USA; a15 Medical Genetics Research Center, Faculty of Medicine, Mashhad University of Medical Sciences, Mashhad, Iran; a16 GeneDx, Gaithersburg, Maryland, USA; a17 Clinical Genetics Department, Human Genetics and Genome Research Division, National Research Centre, Cairo 12311, Egypt; a18 Department of Molecular and Human Genetics, Baylor College of Medicine, Houston, TX, USA; a19 Department of Pediatrics, University of Wisconsin Hospitals and Clinics, Madison, WI, USA; a20 Genetics and Molecular Biology Branch, National Human Genome Research Institute, National Institutes of Health, Bethesda, Maryland, USA

Sir,

We read with great interest the article by Perez *et al.* (2018) on the emerging condition of intellectual disability caused by biallelic pathogenic variants in *RSRC1* (arginine and serine rich coiled-coil 1). Previous genome wide association studies (GWAS) by Potkin *et al.* (2009, 2010) had suggested a possible involvement of *RSRC1* in non-syndromic intellectual disability and gene regulatory networks in schizophrenia. However, the results of these studies remained elusive and were not confirmed by a subsequent GWAS study by Schizophrenia Working Group of the Psychiatric Genomics Consortium (2014). Meanwhile, Berndt *et al.* (2013) identified *RSRC1* as a new locus influencing height through a genome-wide meta-analysis. The first family in which a homozygous *RSRC1* variant clearly segregated with non-syndromic intellectual disability was only recently reported by Maddirevula *et al.* (2018). A possible role of *RSRC1* in major depressive disorder has been further suggested by a meta-analysis on three large GWAS performed by Li *et al.* (2018).

Serine and arginine-rich (SR) proteins are evolutionary conserved co-regulators of constitutive and alternative pre-mRNA splicing. The longest *RSRC1* transcript (NM_001271838.1) includes 10 exons (Fig. 1A) and encodes a 334-amino-acid SR-related protein of 53 kDa (SRrp53) (Fig. 1B), localized to the nuclear speckled domain. Interacting with other splicing regulators, RSRC1 plays a relevant role in the second step of pre-mRNA splicing. In addition, it could be involved in post-splicing mRNA processing, shuttling between the nucleus and cytoplasm (Cazalla *et al.*, 2005). RSRC1 further promotes PIAS1-mediated SUMOylation of the pleiotropic transcription factor oestrogen receptor b (ERb), acting as transcriptional regulator (Chen *et al.*, 2015).

**Figure 1:**
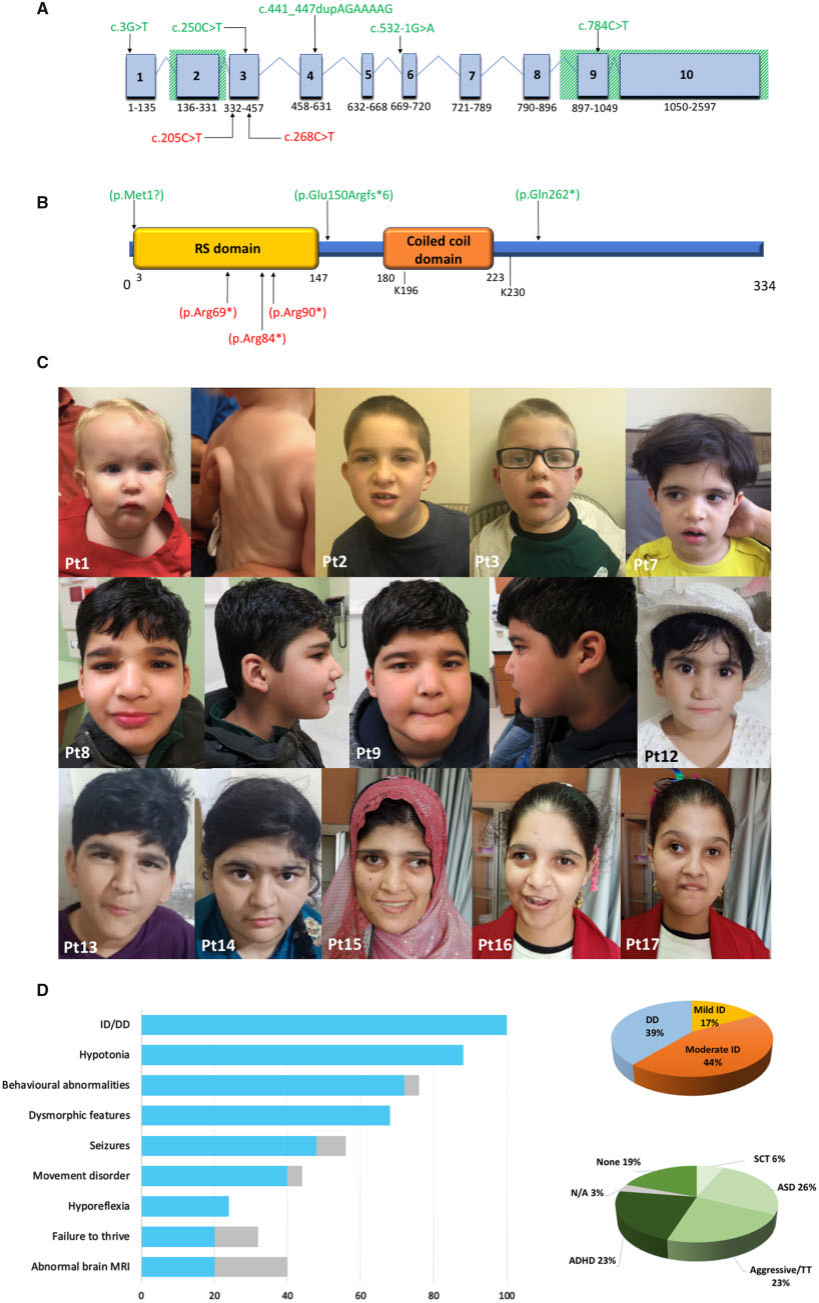
**Genetic findings and clinical pictures of *RSRC1* patients.** (**A**) Schematic diagram of the longer *RSRC1* transcript (NM_001271838.1) consisting of 2597 nucleotides in 10 exons. The deletions encompassing exons 2 and 9–10 are represented by diagonal green lines. Single nucleotide variants are shown in red (previously reported patients) or in green (this study). (**B**) The RSRC1 protein (NP_001258767.1) consists of 334 amino acids encompassing an RS domain rich in arginine (R) and serine (S) that mediates the interactions with other RS-rich proteins involved in splicing regulation (SF2/ASF and U2AF35) and a coiled coil domain required for RSRC1/ERβ interaction as well as the enhancement of ERβ SUMOylation. The residues K196 and K 230 are necessary for RSRC1 SUMOylation by SUMO1 and the E3 ligases PIAS1 and PIAS3. Pathogenic amino acid changes reported in previous papers and identified in this study are shown in red and green, respectively. (**C**) Sequential pictures from selected patients. Patients from the Amish family (Patients 1–3) show prominent forehead, deep set eyes, depressed nasal bridge, protruding ears, and overbite with drooling. Redundant skin is evident in Patient 1. The Persian patient (Patient 7) shows straight eyebrows with mild synophrys, deep set eyes, and protruding ears. In the first Pakistani family (Patients 8 and 9) dysmorphic features include straight eyebrows with mild medial flaring, deep set eyes, wide nasal base, short philtrum, uplifted earlobes, and prominent chin. Subjects from the second Pakistani family (Patients 12–14) also show straight eyebrows with mild synophrys and deep set eyes, in addition to protruding ears with uplifted lobes (Patient 14). Patients from the Egyptian family (Patients 15–17) show thick eyebrows with medial sparing, deep set eyes, and prominent columella. (**D**) Graphic illustrations of the most common clinical findings in *RSRC1* patients in our cohort: the bar graph shows the percent distribution of the cardinal features of *RSRC1*-related intellectual disability, with the grey lines representing not available data; the pie charts show the percentage distribution of developmental delay/intellectual disability and the different behavioural abnormalities observed in *RSRC1* patients. Gene transcript and protein details available at: https://www.ensembl.org (RSRC1-215, transcript ID ENST00000611884.5), https://www.nextprot.org (NX_Q96IZ7), https://www.uniprot.org (Q96IZ7), https://www.proteomicsdb.org (Q96IZ7). ADHD = attention deficit hyperactivity disorder; ASD = autism spectrum disorder; DD = developmental delay; ID = intellectual disability; N/A = not available; Pt = patient; SCT = sluggish cognitive tempo; TT = temper tantrums.

The involvement of *RSRC1* in cancer was first suggested by the identification of the recurrent *PTPLB*-*RSRC1* in-frame gene fusion in nasopharyngeal carcinoma by Valouev *et al.* (2014). Teplyuk *et al.* (2016) showed that *RSRC1* is a target gene of the microRNA‐10b (miR‐10b), an oncogenic microRNA that is highly expressed in glioblastoma and represents a candidate for the development of targeted therapies. More recently, several studies have implicated RSRC1 in cancer predisposition and progression. The *RSRC1* intronic polymorphism rs6441201 G > A has been associated with neuroblastoma susceptibility (McDaniel *et al.*, 2017; Tang *et al.*, 2018). A possible role of RSRC1 in non-small-cell lung cancer tumorigenesis and progression has been hypothesized based on the data from RNA sequencing data of tumour-educated platelets (Sheng *et al.*, 2018). RSRC1 has been also shown to suppress gastric cancer cell proliferation and migration through the regulation of PTEN expression, acting as tumour suppressor (Yu *et al.*, 2019).

The first *RSRC1* pathogenic variant segregating with intellectual disability was reported by Maddirevula *et al.* (2018) in three affected siblings from a consanguineous Malaysian family (Supplementary Table 3). The homozygous truncating variant c.268C>T (p.Arg90*) (NM_001271838.1) resulted in a full loss of function, causing the natural knockout of the gene. All the reported patients showed developmental delay and variable degree of intellectual disability. One subject also suffered from febrile seizures. Brain MRI was normal in the 10-year-old male (Patient 1), whereas temporal lobe atrophy was found in his 4-year-old brother (Patient 2). No distinctive neurological features or facial dysmorphism were observed. More recently, Perez *et al.* (2018) reported five further individuals from consanguineous Bedouin kindred with early developmental delay, intellectual disability, hypotonia, behavioural abnormalities, and mild facial dysmorphic features. Brain MRI was normal (Supplementary Table 3). Exome sequencing revealed the homozygous c.205C>T (p.Arg69*) in *RSRC1* (NM_001271838.1) in all patients, a nonsense variant leading to nonsense-mediated mRNA decay. *RSRC1* knock-down SH-SY5Y cells showed impaired alternative splicing. Specific differential expression of genes associated with intellectual disability, hypotonia, schizophrenia, and dementia was also observed, supporting the pivotal role of *RSRC1* in transcriptional regulation (Perez *et al.*, 2018).

We report 17 additional subjects from seven consanguineous families with intellectual disability, behavioural abnormalities, and facial dysmorphism, harbouring homozygous *RSRC1* loss-of-function variants (Table 1 and Supplementary Table 1). The families were of different ancestries (European/Middle Eastern, Saudi, Egyptian, Old Order Amish, Pakistani, and Persian) (Supplementary Fig. 1). The collaboration among the involved study centres was managed through GeneMatcher (Sobreira *et al.*, 2015). After informed consent was obtained from the parents, photographic material was collected and genetic testing through exome sequencing was performed. Genetic methods are provided in detail in the Supplementary material. Trio-exome sequencing was conducted in Patient 10, the family quartet was sequenced for Patients 8 and 9, and proband-exome sequencing was performed in all the remaining individuals, followed by variants validation through Sanger sequencing. No relevant single nucleotide variant was identified in the siblings of Amish ancestry (Patients 1–4), who were further studied through comparative genomic hybridization (CGH) array. Five *RSRC1* sequence variants (including an intragenic duplication) and two partial deletions were identified.

**Table I:** Summary of genetic findings and clinical features of *RSRC1* patients

Family	Family I (Amish)				Family II (Persian)			Family III (Pakistani)		Family IV (Saudi)	Family V (EUR/ ME)	Family VI (Pakistani)			Family VII (Egyptian)			Maddirevula *et al.*^2018 ^ (Malaysian)	Perez *et al.*^2018 ^ (Bedouin)
Patient	1(II-2)	2(II-3)	3(II-4)	4(II-5)	5(II-3)	6(II-1)	7(II-2)	8(II-2)	9(II-1)	10(II-2)	11(II-1)	12(II-6)	13(II-4)	14(II-3)	15(II-5)	16 (II-8)	17(II-9)	3 patients	5 patients
Age/sex	3 y/F	6 y/M	5 y/M	11 mo/M	6 y/M	14 y/F	3 y/F	9 y/M	11 y/M	4 y/M	16 y/F	6 y/F	15 y/M	16 y/F	22 y/F	16 y/F	12 y/F	4–10 y/2M, 1F	0.5–8 y/3F, 2M
Homozygous RSRC1 variants [NM_001271838.1]	c.158,256,914_158,338,237del^a^				c.784 C>T (p.Gln262*)			c.157,839,811_ 157,840, 314del^b^		c.250 C>T (p.Arg84*)	c.441_447dupAGAAAAG (p.Glu150Argfs*6)	c.532-1 G>A			c.3G>T(p.Met1?)			c.268C>T (p.Arg90*)	c.205C>T (p.Arg69*)
Consanguinity	+	+	+	+	+	+	+	+	+	+	+	+	+	+	+	+	+	+ (3)	+ (5)
Dysmorphic features	+	+	+	+	+	+	+	–	–	+	+	+	+	+	–	–	–	–	+ (5)
Global DD/ID	+	+	+	+	++	++	+	++	++	+	+	++	++	++	++	++	++	+ (5)	+/++ (5)
Speech delay	N/A	+	+	+	N/A	N/A	N/A	N/A	N/A	+	+	+	+	–	–	+	–	+ (1)	+ (4)
Behavioural abnormalities	–	ASD	ADHD, ASD	–	ASD	ASD	ASD	ADHD	ADHD	–	SCT	Agg	Agg	SCT	ASD	Agg	ASD	–	TT (4), ASD (1), ADHD (4)
Hypotonia	+++	++	+	++	++	++	++	+	+	+	++	+	+	+	+	+	+	–	+ (5)
Movement disorders	–	Gait ataxia	Gait ataxia	–	Gait ataxia	Gait ataxia	–	–	–	Truncal ataxia	Brady-kinesia, ataxic gait	–	–	–	–	–	–	–	Fine motor impairment (4)
Seizures	–	–	–	–	N/A	–	FS, GTCS	FS	–	GTCS	–	FS, GTCS	FS, GTCS	N/A	–	–	FS	FS (1)	FS (5), epilepsy (1)
Musculo-skeletal abnormalities	–	PP, CV	PP, CV	–	–	–	–	PP	PP	–	PP, CV, short toes	PP, short fifth toes	CV, genu valgum	PP	PP	PP	–	Foot deformities (2)	–
Brain MRI	N	PSS	PSS	N/A	MCA	N	N	N	N	Left temporo- parietal atrophy	N	N/A	N/A	N/A	N	N	N	Temporal lobes atrophy (1)	N (5)

ADHD = attention deficit hyperactivity disorder; Agg = aggressive; ASD = autism spectrum disorder; CV = cubitus valgus; DD = developmental delay; EUR = European; FS = febrile seizures; GTCS = generalized tonic-clonic seizures; ID = intellectual disability (+, mild; ++, moderate); MCA = mild cerebral atrophy; ME = Middle Eastern; mo = months; N = normal; N/A = not available; PP = pes planus; PSS = prominent subarachnoid spaces; SCT = sluggish cognitive tempo; y = years.

^a^ hg19; 81-kb deletion encompassing exons 9–10 of *RSRC1* and whole *MLF1*.

^b^ hg19; 500-bp deletion encompassing exon 2 of *RSRC1*.

In all the enrolled subjects, global psychomotor developmental delay and mild-to-moderate intellectual disability were observed. Twelve patients were diagnosed with variable behavioural disorders (Table 1 and Supplementary Table 1). Neurological examination revealed generalized hypotonia in all patients and five of them had a history of hypotonia at birth. Decreased deep tendon reflexes were found in six patients (35%). Five patients showed gait ataxia, which was associated with bradykinesia in Patient 11. Truncal ataxia was observed in Patient 10. Seizures occurred in six individuals (35%), including four cases of febrile seizures. Afebrile generalized tonic-clonic seizures in addition to febrile seizures were observed in Patients 7, 12, and 13. Patient 10 experienced three episodes of generalized tonic-clonic seizures before becoming seizure-free. His EEG showed occasional epileptiform discharges in the centrofrontal and in the left parietocentral regions during sleep. No recurrent epileptic phenotype or peculiar EEG features were recognizable in our cohort. Psychomotor regression was not observed in any case. The most common dysmorphic features were deep-set eyes, broad nasal base, and ogival palate (Fig. 1C). Associated congenital anomalies were extremely variable, ranging from simple pes planus to redundant skin. Other isolated clinical features included mitral valve prolapse, recurrent respiratory infections in the first year of life, and tracheomalacia. When available, brain MRI did not reveal any distinctive finding. Non-specific mild cerebral atrophy was observed in Patients 5 and 6, whereas enlargement of subarachnoid spaces was found in Patients 2 and 3. Delayed myelination, dysmorphic lateral ventricles, and unilateral focal polymicrogyria were observed in Patient 10.

In our cohort, we identified homozygous *RSRC1* variants or deletions leading to loss of function (Table 1 and Supplementary Table 1). Variants’ nomenclatures are given with reference to the *RSRC1* transcript NM_001271838.1. The four Amish siblings (Patients 1–4) carried an 81-kb deletion encompassing exons 9–10 of *RSRC1* and the entire *MLF1* gene. Although *MLF1* is involved in haematopoiesis and leukemogenesis, a possible pathogenic role for this gene in some of the dysmorphic features and congenital anomalies in the Amish patients cannot be excluded (Yoneda-Kato *et al.*, 1996; Nakamae *et al.*, 2017). A second 500-bp deletion involving exon 2 of *RSRC1* was identified in the siblings from the Pakistani family (Patients 8 and 9). Both these rearrangements are novel and are not reported in ClinVar and DECIPHER databases (Supplementary Table 1). The three patients from the second Pakistani family (Patients 12–14) carried the splicing variant c.532-1G>A. This amino acid change is predicted to cause aberrant splicing through the alteration of the splice acceptor site at exon 6 of *RSRC1* (Supplementary Table 3). In the remaining families, homozygous stop-gain or start-loss variants were identified. The 16-year-old female of European ancestry (Patient 11) harboured the intragenic duplication c.441_447dupAGAAAAG (p.Glu150Argfs*6). The stop-gain variants c.784C>T (p.Gln262*) and c.250C>T (p.Arg84*) were identified in the siblings from the Persian (Patients 5–7) and Middle East (Patient 10) families, respectively. In the Egyptian family (Patients 15–17), the variant c.3G>T (p.Met1?) causing a start loss and leading to full loss of function (null variant) was found.

All the variants fully segregated with the phenotype and were not found in the most common genome databases, including the Genome Aggregation Database (gnomAD), Iranome, Greater Middle East Variome Project (GME Variome), and our database of 10 000 in-house control exomes. The only exception was the splicing variant c.532-1G>A, which was observed in heterozygous state in 3 of 166 052 in gnomAD (allele frequency 0.00001806). Furthermore, the start loss variant affecting the same nucleotide of the c.3G>T (p.Met1?) variant observed in our Egyptian family is reported in heterozygous state in gnomAD (genomes allele frequency 0.00003186). However, neither variant has been reported in homozygous state in healthy individuals. The analysis of sequence conservation through Genomic Evolutionary Rate Profiling (GERP) score revealed good conservation of the affected residues and *in silico* prediction analysis through CADD score calculation revealed high-deleterious scores (Supplementary Table 3). All variants were predicted to be damaging or likely damaging by several bioinformatic tools (e.g. SIFT, MutationTaster, and Human Splice Finder) and were classified as pathogenic (class 5) or likely pathogenic (class 4) according to the American College of Medical Genetics and Genomics (ACMG) guidelines (Richards *et al.*, 2015).

This study supports the idea that *RSRC1* pathogenic variants cause a non-syndromic disorder characterized by mild-to-moderate intellectual disability, generalized hypotonia, and variable neurological and behavioural features (Fig. 1D). Despite a few minor dysmorphic features being observed (especially deep-set eyes and broad nasal base), a consistent recurrent facial gestalt could not be recognized. Furthermore, the extremely variable associated non-neurological features were not suggestive of a syndromic condition. Even though seizures are common in *RSRC1* patients, most of them only suffer from febrile seizures, lacking a distinctive epileptic phenotype. Definite epileptic seizures were only diagnosed in Patient 12 and in a case reported by Perez *et al.* (2018). This patient suffered from focal seizures with impaired awareness occasionally progressing to tonic-clonic and was treated with valproic acid. Behavioural abnormalities are frequent, although extremely variable and ranging from attention deficit hyperactivity disorder (ADHD) to autism spectrum disorder (ASD) (Fig. 1D). Besides the non-specific neuroradiological abnormalities observed in our patients, bilateral symmetric temporal lobe atrophy has been described in a single case by Maddirevula *et al.* (2018). Even though further neuroimaging studies will be necessary, the limited data available support the lack of a peculiar neuroradiological phenotype. According to these observations, biallelic *RSRC1* variants should be considered the cause of a non-syndromic intellectual disability mainly associated with generalized hypotonia and behavioural disturbances. Facial dysmorphism and other minor clinical features have limited diagnostic relevance, likewise neuroimaging is of limited value.

Thanks to the remarkable advances in gene discovery achieved through exome sequencing, the large group of known genes causing non-syndromic intellectual disability is rapidly expanding with relevant impact on diagnosis and patient management. Our findings support the pathogenic role of biallelic loss-of-function *RSRC1* variants in autosomal recessive intellectual disability, in addition to contributing to the phenotypic delineation of this emerging condition. Global developmental delay, mild-to-moderate intellectual disability, behavioural abnormalities, and generalized hypotonia represent the cardinal features of *RSRC1*-related intellectual disability. Other neurological features may be less frequently observed, especially hyporeflexia and febrile seizures. Further studies will help clarify the possible role of neuroimaging in the diagnostic process. In conclusion, we suggest that the involvement of *RSRC1* should be considered in the differential diagnosis in intellectually disabled children with hypotonia and behavioural disturbances, and that *RSRC1* should be included in next generation sequencing (NGS) panels for intellectual disability.

## Data availability

Data sharing is not applicable to this article, as no new data were created or analysed in this study.

## Web resources

The following URLs were used for data presented herein:

ClinVar; https://www.ncbi.nlm.nih.gov/clinvar

Combined Annotation Dependent Depletion (CADD); http://cadd.gs.washington.edu

DECIPHER; https://decipher.sanger.ac.uk

Ensembl; https://www.ensembl.org/index.html

Gene Cards; http://www.genecards.org

Gene Matcher; http://www.genematcher.org

Genome Aggregation Database (GnomAD); http://gnomad.broadinstitute.org

Greater Middle East (GME) Variome Project; http://igm.ucsd.edu/gme/

Human Splice Finder; http://www.umd.be/HSF

Iranome; http://www.iranome.ir

Mutalyzer; https://mutalyzer.nl

Mutation Taster; http://www.mutationtaster.org

NeXtProt; https://www.nextprot.org

Online Mendelian Inheritance in Man; http://www.ncbi.nlm.nih.gov/Omim

Proteomics DB; https://www.proteomicsdb.org

PubMed; http://www.ncbi.nlm.nih.gov/pubmed

RefSeq; https://www.ncbi.nlm.nih.gov/refseq

SIFT; https://sift.bii.a-star.edu.sg

The 1000 Genomes Browser; http://browser.1000genomes.org/index.html

The Greater Middle East (GME) Variome Project; http://igm.ucsd.edu/gme/index.php

UniProt; https://www.uniprot.org

UCSC Human Genome Database; http://www.genome.ucsc.edu

Varsome; https://varsome.com

## Supplementary Material

awaa070_Supplementary_Data
