# Supplementary material for: RSRC1 loss-of-function variants cause mild to moderate autosomal recessive intellectual disability
Source: Brain. 2020 Mar 30;143(4):e31. doi: 10.1093/brain/awaa070 (PMC7174030; doi:10.1093/brain/awaa070)
Supplement: awaa070_Supplementary_Data [file awaa070_supplementary_data.pdf]

Supplementary information

Content

1. **Supplementary Genetic Methods**
2. **Supplementary Tables**
3. **Supplementary Figure**
4. **Supplementary References**

1. Supplementary Genetic Methods

DNA isolation

Genomic DNA was extracted from whole blood from family members using DNeasy Blood & Tissue kit (Qiagen, Hilden Germany), according to the manufacturer's instructions.

Array-CGH

Array-CGH was performed for the siblings of Amish ancestry (pts 1-4) using a 180 k microarray according to the manufacturer's instructions (Agilent Technologies, Santa Clara, CA, USA). Agilent Cytogenomics software v3.3 was then used to analyse the results.

Exome Sequencing

After standard DNA extraction, exome sequencing (ES) was performed in all families. In the family of Middle Eastern ancestry (patient 10) trio WES was performed, whereas the remaining patients were studied through proband-ES. WES was performed as previously described by Kariminejad et al. (2016) and Yang et al (2014). The quality of the sequence reads was assessed by generating QC statistics with FastQC (http://www.bioinformatics.bbsrc.ac.uk/projects/fastqc). BWA with default parameters was

used for the read alignment to the reference human genome (hg19, UCSC assembly, February 2009) (Li and Durbin, 2009). According to established best practices (DePristo et al., 2011), HaplotypeCaller algorithm in the GATK package was used for quality score recalibration, indel realignment, and variant calling (McKenna et al., 2010). Subsequently, variants were annotated with ANNOVAR (Wang et al., 2010) and then filtered for population genetics and impact on the protein function. Variants with a minor allele frequency (MAF) >0.01 in control databases (internal database of 10000 exomes, GnomAD, GME, Iranome, and Ensembl) were excluded. Subsequently, the variant analysis was performed through bioinformatic prediction tools (SIFT, Mutation Taster, and Human Splice Finder) and were further filtered on the basis of their CADD and GERP scores were also calculated. Eventually, the most interesting candidate variants were confirmed through Sanger sequencing.

2. Supplementary Tables

Supplementary Table I Genetic findings and clinical features of *RSRC1* patients.

	Family I				Family II			Family III		Family IV	Family V
	**Pt 1 (II-2)**	**Pt 2 (II-3)**	**Pt 3 (II-4)**	**Pt 4 (II-5)**	**Pt 5 (II-3)**	**Pt 6 (II-1)**	**Pt 7 (II-2)**	**Pt 8 (II-2)**	**Pt 9 (II-1)**	**Pt 10 (II-2)**	**Pt 11 (II-1)**
**Age; sex**	3 y; F	6 y; M	5 y; M	11 mo; M	6 y; M	14 y; F	3 y; F	9 y; M	11 y; M	4 y; M	16 y; F
**Ancestry**	Amish	Amish	Amish	Amish	Persian	Persian	Persian	Pakistani	Pakistani	Saudi	EUR/ME
***RSRC1* variants [NM_001271838.1]**	c.158,256,914 _158,338,237del (hom)†	c.158,256,914 _158,338,237del (hom)†	c.158,256,914 _158,338,237del (hom)†	c.158,256,914 _158,338,237del (hom)†	c.784C>T (p.Gln262*) (hom)	c.784C>T (p.Gln262*) (hom)	c.784C>T (p.Gln262*) (hom)	c.157,839,811 _157,840,314del (hom)‡	c.157,839,811 _157,840,314del (hom)‡	c.250C>T (p.Arg84*) (hom)	c.441_447dup AGAAAAG (p.Glu150Argfs*6) (hom)
**Consanguinity**	+	+	+	+	+	+	+	+	+	+	+
**Previous miscarriages**	+	-	-	-	-	-	-	-	-	-	-
**Birth history**	Floppy	Normal	Normal	Normal	Floppy	Floppy	Floppy	Normal	Normal	Floppy	Floppy
**FTT**	+	+	-	-	-	-	-	-	-	+	-
**Dysmorphic features**	+	+	+	+	+	+	+	-	-	+	+
**Global DD/ ID (degree)**	+	+/ + (mild)	+	+	+/ + (mod)	+/ + (mod)	+	+/ + (mod)	+/ + (mod)	+	+/ + (mild)
**Speech delay**	N/A	+	+	+	N/A	N/A	N/A	N/A	N/A	+	+
**Behavioral abnormalities**	-	ASD	ADHD, ASD	-	ASD	ASD	ASD	ADHD	ADHD	-	SCT
**Hypotonia**	+++	++	+	++	++	++	++	+	+	+	++
**Hyporeflexia**	+	-	-	-	+	+	+	-	-	+	-
**Movement disorders**	-	Gait ataxia	Gait ataxia	-	Gait ataxia	Gait ataxia (until 4 y)	-	-	-	Truncal ataxia	Bradykinesia, ataxic gait
**Seizures**	-	-	-	-	N/A	-	FS, GTCS	FS	-	GTCS, abnormal EEG	-
**Eye findings**	Strabismus	-	Strabismus	-	Strabismus	-	-	-	-	Strabismus, ptosis	Strabismus
**Dysphagia/ GI reflux**	+/+	-/-	-/-	-/-	-/-	-/-	-/-	-/-	-/-	+/+	-/+
**Musculoskeletal abnormalities**	-	PP, cubitus valgus	PP, cubitus valgus	-	-	-	-	PP	PP	-	PP, cubitus valgus, short toes
**Other clinical features**	Weak cry, drooling, redundant skin, skin dimples	Drooling, redundant skin, skin dimples, incontinence	Drooling, redundant skin, skin dimples, incontinence	Redundant skin, skin dimples	Weak cry	Weak cry, drooling, urinary and bowel incontinence	Weak cry, drooling	-	-	Neonatal intestinal obstruction, tracheo-malacia	Mitral valve prolapse
**Brain MRI**	Normal	Prominent subarachnoid spaces	Prominent subarachnoid spaces	N/A	Mild cerebral atrophy	Normal	Normal	Normal	Normal	Left temporo-parietal atrophy	Normal

Supplementary Table I Genetic findings and clinical features of *RSRC1* patients (Continued).

	Family VI			Family VII			Maddirevula *et al*, 2018	Perez *et al*, 2018
	Pt 12 (II-6)	Pt 13 (II-4)	Pt 14 (II-3)	Pt 15 (II-5)	Pt 16 (II-8)	Pt 17 (II-9)	3 pts	5 pts
**Age; sex**	6 y; F	15 y; M	16 y; F	22 y; F	16 y; F	12 y; F	4-10 y; 2M-1F	0.5-8 y; 3F-2M
**Ancestry**	Pakistani	Pakistani	Pakistani	Egyptian	Egyptian	Egyptian	Malaysian	Bedouin
***RSRC1* variants [NM_001271838.1]**	c.532-1G>A (hom)	c.532-1G>A (hom)	c.532-1G>A (hom)	c.3G>T (p.Met1?) (hom)	c.3G>T (p.Met1?) (hom)	c.3G>T (p.Met1?) (hom)	c.268C>T (p.Arg90*) (hom)	c.205C>T (p.Arg69*) (hom)
**Consanguinity**	+	+	+	+	+	+	+ (3)	+ (5)
**Previous miscarriages**	+ (2)	+ (2)	+ (2)	+(1)	+ (1)	+ (1)	-	-
**Birth history**	Normal	Normal	Normal	Normal	Normal	Normal	N/A	Normal
**FTT**	N/A	N/A	N/A	No	No	No	+ (1)	-
**Dysmorphic features**	+	+	+	-	-	-	-	+ (5)
**Global DD/ ID (degree)**	+/ + (mod)	+/ + (mod)	+/ + (mod)	+/ + (mod)	+/ + (mod)	+/ + (mod)	+ (3)/ + (mild) (2)	+ (5)/ + (mild-mod) (2)
**Speech delay**	+	+	-	-	+	-	+ (1)	+ (4)
**Behavioural abnormalities**	Aggressive behaviour	Aggressive behaviour	SCT	ASD	Aggressive behaviour	ASD	-	TT (4), ASD (1), ADHD (4)
**Hypotonia**	+	+	+	+	+	+	-	+ (5)
**Hyporeflexia**	+	-	-	-	-	-	-	-
**Movement disorders**	-	-	-	-	-	-	-	Fine motor impairment (4)
**Seizures**	FS, GTCS	FS, GTCS	N/A	-	-	FS	FS (1)	FS (5), epilepsy (1)
**Eye findings**	-	-	-	Normal	Normal	Normal	-	-
**Dysphagia/ Reflux**	+/-	+/+	-/-	-/-	-/-	+/-	-	-
**Musculoskeletal abnormalities**	PP, sandal gap, short 5th toes	Cubitus valgus, genu valgum	PP	PP	PP	-	Feet deformities (2)	-
**Other clinical features**	Redundant skin	Redundant skin	Redundant skin, urinary and bowel incontinence	-	-	-	Recurrent respiratory infections (1)	Incontinence (4), microcephaly (1)
**Brain MRI**	N/A	N/A	N/A	Normal	Normal	Normal	Temporal lobes atrophy (1)	Normal (5)

ADHD = attention deficit hyperactivity disorder; ASD = autism spectrum disorder; DD = developmental delay; EUR = European; FS = febrile seizures; FTT = failure to thrive; GTCS = generalized tonic-clonic seizures; hom = homozygous; ID = intellectual disability; ME = Middle Eastern; mo = months; mod = moderate; N/A = not available; PP = pes planus; Pt = patient; SCT = sluggish cognitive tempo; y = years. †hg19; 81-kb deletion encompassing exons 9-10 of *RSRC1* and whole *MLF1*. ‡hg19; 500-bp deletion encompassing exon 2 of *RSRC1*.

Supplementary Table 2 Frequency of the rearrangements involving *RSRC1* reported in this study.

RSRC1 Deletion	Decipher	ClinVar
**c.157,839,811_157,840,314del**	-	-
**c.158,256,914_158,338,237del**	-	-

Supplementary Table 3 Frequency and predicted effect of all the reported *RSRC1* variants.

***RSRC1* variant [NM_001271838.1]**	**g. (hg19)**	**Internal database‡**	**GeneDx database**	**ExAC/ gnomAD**	**GME**	**Iranome**	**Ensembl**	**ClinVar**	**SIFT**	**Mutation Taster**	**Human splice finder**	**GERP score**	**CADD score**	**ACMG class**
**c.3G>T p.(Met1Ile)**	g.157839896G>T	-	-	-	-	-	-	-	Damaging	Disease causing	N/A	5.66	25	Pathogenic (PVS1, PM2, PP3)
**c.205C>T (p.Arg69*)**	g.157841665C>T	-	1/130,874	-	-	-	rs868818936	Pathogenic	Damaging	Disease causing	N/A	4.17	39	Pathogenic (PVS1, PM2, PP3)
**c.250C>T (p.Arg84*)**	g.157841710C>T	-	1/130,874	-	-	-	rs267599665	-	Damaging	Disease causing	N/A	5.39	39	Pathogenic (PVS1, PM2, PP3)
**c.268C>T (p.Arg90*)**	g.157841728C>T	-	1/183,384	-	-	-	rs1183090232	Pathogenic	Damaging	Disease causing	N/A	3.61	37	Pathogenic (PVS1, PM2, PP3)
**c.441_447dupAGAAAAG (p.Glu150Argfs*6)**	g.157920981_157920987dupAGAAAAG	-	-	-	-	-	-	-	N/A	Disease causing	N/A	2.13	N/A	Likely pathogenic (PVS1, PM2)
**c.532-1G>A**	g.158072645G>A	-	1/174,424	0.000012 (2 het)	-	-	rs754550509	-	N/A	Disease causing	Most probably affecting splicing†	5.88	32	Pathogenic (PVS1, PM2, PP3)
**c.784C>T (p.Gln262*)**	g.158261148C>T	-	-	-	-	-	-	-	Damaging	Disease causing	N/A	4.06	35	Likely pathogenic (PVS1, PM2)

CADD = Combined Annotation Dependent Depletion; GERP = Genomic Evolutionary Rate Profiling; gnomAD = Genome Aggregation Database; GME = Greater Middle East (GME) Variome Project; N/A = not applicable; PM2: Pathogenic Moderate 2; PP3 = Pathogenic Supporting 3; PVS1 = Pathogenic Very Strong 1; SIFT = Sorting Intolerant From Tolerant. †Alteration of the WT acceptor site. ‡Database of 10,000 in-house control exomes.

Supplementary Table 4 Summary of genetic findings and clinical features of previously reported *RSRC1* patients.

	**Maddirevula 2018, pt1**	**Maddirevula 2018, pt2**	**Maddirevula 2018, pt3**	**Perez 2018 II-6**	**Perez 2018 II-7**	**Perez 2018 II-10**	**Perez 2018 II-11**	**Perez 2018 II-12**
**Age; sex**	10 y; M	4 y; M	7 y; F	8 y; F	7 y; M	3.5 y; F	5 y; F	0.5 y; M
**Ancestry**	Malaysian	Malaysian	Malaysian	Bedouin	Bedouin	Bedouin	Bedouin	Bedouin
***RSRC1* variants [NM_001271838.1]**	c.268C>T: (p.Arg90*) (hom)	c.268C>T: (p.Arg90*) (hom)	c.268C>T: (p.Arg90*) (hom)	c.205C>T; (p.Arg69*) (hom)	c.205C>T; (p.Arg69*) (hom)	c.205C>T; (p.Arg69*) (hom)	c.205C>T; (p.Arg69*) (hom)	c.205C>T; (p.Arg69*) (hom)
**Consanguinity**	+	+	+	+	+	+	+	+
**Previous miscarriages**	-	-	-	-	-	-	-	-
**Birth history**	N/A	N/A	N/A	Normal	Normal	Normal	Normal	Normal
**FTT**	+	-	-	-	-	-	-	-
**Dysmorphic features**	-	-	-	+	+	+	+	+
**Global DD/ ID (degree)**	+/ + (mild)	+	+/ + (mild)	+/ + (mild-mod)	+/ + (mild-mod)	+	+	+
**Speech delay**	N/A	+	N/A	+	+	+	+	N/A
**Behavioural abnormalities**	-	-	-	TT, ASD, ADHD	TT, ADHD	TT, ADHD	TT, ADHD	N/A
**Hypotonia**	-	-	-	+	+	+	+	+
**Hyporeflexia**	-	-	-	-	-	-	-	-
**Movement disorders**	-	-	-	Fine motor impairment	Fine motor impairment	Fine motor impairment	Fine motor impairment	N/A
**Seizures**	-	FS	-	FS	FS	FS, epilepsy	FS	FS
**Eye findings**	-	-	-	-	-	-	-	-
**Dysphagia/ Reflux**	-	-	-	-	-	-	-	-
**Musculoskeletal abnormalities**	-	Bilateral calcaneo-varus deformity	Metatarsus valgus	-	-	-	-	-
**Other clinical features**	-	Recurrent respiratory infections	-	Incontinence, microcephaly	Incontinence	Incontinence	Incontinence	N/A
**Brain MRI**	Normal	Temporal lobes atrophy	N/A	Normal	Normal	Normal	Normal	Normal

ADHD = attention deficit hyperactivity disorder; ASD = autism spectrum disorder; DD = developmental delay; FS = febrile seizures; FTT = failure to thrive; hom = homozygous; ID = intellectual disability; mod = moderate; N/A = not available; pt = patient; TT = temper tantrums; y = years.

3. **<u>Supplementary Figure</u>**

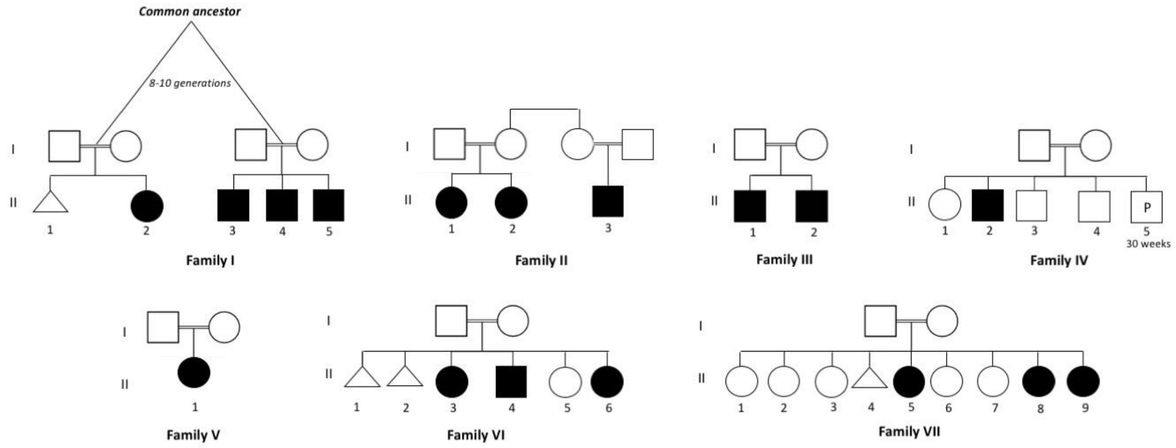

Supplementary Figure 1 Pedigrees of the reported families. Displayed symbols make reference to the Standardized Human Pedigree Nomenclature (Bennett *et al.*, 2008). Family I is an extended Amish family with a common ancestor composed of two distantly related families of different Amish *demes* (Lancaster and Indiana).

4. Supplementary References

Bennett RL, French KS, Resta RG, Doyle DL. Standardized human pedigree nomenclature: update and assessment of the recommendations of the National Society of Genetic Counselors. J Genet Couns 2008; 17(5): 424–33.

DePristo MA, Banks E, Poplin R, Garimella KV, Maguire JR, Hartl C, et al. A framework for variation discovery and genotyping using next-generation DNA sequencing data. Nat Genet 2011; 43: 491–8.

Li H, Durbin R. Fast and accurate short read alignment with Burrows-Wheeler transform. Bioinformatics 2009; 25: 1754–60.

Kariminejad A, Ghaderi-Sohi S, Hossein-Nejad Nedai H, Varasteh V, Moslemi AR, Tajsharghi H.

Lethal multiple pterygium syndrome, the extreme end of the RYR1 spectrum. BMC Musculoskelet Disord 2016; 17: 109.

McKenna A, Hanna M, Banks E, Sivachenko A, Cibulskis K, Kernytsky A, et al. The Genome Analysis Toolkit: a MapReduce framework for analyzing next-generation DNA sequencing data. Genome Res 2010; 20: 1297–303.

Wang K, Li M, Hakonarson H. ANNOVAR: functional annotation of genetic variants from high-throughput sequencing data. Nucleic Acids Res 2010; 38: e164.

Yang Y, Muzny DM, Xia F, Niu Z, Person R, Ding Y, et al. Molecular findings among patients referred for clinical whole-exome sequencing. JAMA 2014; 312(18): 1870–9.
